# Graphene Family Materials in Bone Tissue Regeneration: Perspectives and Challenges

**DOI:** 10.1186/s11671-018-2694-z

**Published:** 2018-09-18

**Authors:** Xinting Cheng, Qianbing Wan, Xibo Pei

**Affiliations:** 1 State Key Laboratory of Oral Diseases, National Clinical Research Center for Oral Diseases, West China Hospital of Stomatology, Sichuan University, No. 14, Section 3, South Peoples Road, Chengdu, 610041 China; 2 Department of Prosthodontics, West China Hospital of Stomatology, Sichuan University, No. 14, Section 3, South Peoples Road, Chengdu, 610041 China

**Keywords:** Graphene family materials, Bone tissue regeneration, Scaffold, Coating, Guided bone regeneration membrane, Drug delivery system

## Abstract

We have witnessed abundant breakthroughs in research on the bio-applications of graphene family materials in current years. Owing to their nanoscale size, large specific surface area, photoluminescence properties, and antibacterial activity, graphene family materials possess huge potential for bone tissue engineering, drug/gene delivery, and biological sensing/imaging applications. In this review, we retrospect recent progress and achievements in graphene research, as well as critically analyze and discuss the bio-safety and feasibility of various biomedical applications of graphene family materials for bone tissue regeneration.

## Introduction

Victims of severe maxillofacial infection, trauma, tumor, and congenital deformity suffering jaw bone defects, usually require prolonged convalescence. Unlike many other tissues, the bone has an outstanding capability to regenerate when damaged [1, 2]. However, limited self-regenerating capacity of human skeleton makes the reconstruction of large enough or critical-sized bone defect a significant challenge for clinical therapy [3]. In some cases, the severe patients even need extensive bone augmentation surgeries. Current therapies for bone regeneration consist of autograft, allograft, and xenograft [4]. Autologous bone is considered as the “gold standard” bone-grafting material, with the capabilities of osteoconduction, osteoinduction, and osteogenesis, without immunogenicity as well. But the reasons why autograft is still limited to be used in clinic are the risk of donor infection and the long recovery time [5]. Allograft, obtained from another individual, is often considered as the next best option. But the use of allograft presents potential risks, such as dramatically increased risk of infection and immune rejection [4, 6]. Xenograft materials, such as acid-digested demineralized bone matrix and bovine collagen, are easily obtained and manufactured. Now, xenograft is a main approach in clinical practice. But it has low osteoinductive capacities [7]. At present, compared with the bone, there are no available heterologous or synthetic bone substitutes which have superior or even the same biological or mechanical properties [5]. Although these therapies have been proved useful, they suffer from inherent challenges. Hence, an adequate bone regeneration therapy still needs to be researched and developed. Admittedly, bone tissue engineering and regenerative medicine research open avenues for improving outcomes and speeding recovery of patients with bone defect [8]. Tissue-engineered bone constructs have the potential to alleviate the demand arising from the shortage of suitable autograft and allograft materials for augmenting bone healing [9]. To increase the bone volume in bone defects areas, a variety of methods for bone regeneration have been developed, including scaffolds [1, 6], coatings [10], and barrier membranes for guided bone regeneration (GBR) [11, 12].

Currently, the potential of graphene family materials has captured tremendous attention as 2D planar coating or 3D porous scaffolds for the differentiation of various types of stem cells towards neurogenic [13–15], chondrogenic [16, 17], myogenic [18], adipogenic [19], and osteogenic lineages [20, 21]. Thus, graphene family materials are more likely to be a candidate of the choice for next bone regeneration materials. Graphene, defined as a single or few layers of sp^2^-hybridized carbon atoms, firstly was isolated from graphite by Novoselov and Geim in 2004 [22]. With the increasing research interests, materials of the graphene family, including graphene oxide (GO), carboxyl graphene (CXYG), reduced graphene oxide (rGO), and graphene quantum dots (GQDs), are extensively studied. Graphene possesses exceptionally mechanical, conductive, thermal, and optical properties [23–25], which has been widely applied in electronics, biotechnology, and polymer science [26]. It is acknowledged that conductive materials with promising conductivity enhance cellular activities and stimulate bone tissue repair [27, 28], exhibiting good antibacterial activity as well [29]. Graphene oxide (GO) and carboxyl graphene (CXYG) are both derivatives of graphene. Due to the presence of oxygenated functional groups (epoxide, carboxyl, and hydroxyl groups), GO and CXYG have better dispersion in hydrophilic solvents, which is essential for biomedical applications [30, 31]. Reduced graphene oxide (rGO) can be synthesized by reduction of GO with specific reducing agents under certain conditions. Thanks to the reduction of some special π-π chemical interactions, rGO owns certain better physical and chemical properties than graphene and GO [32, 33]. The raw material of graphene quantum dots (GQDs) is GO. GQDs have strong quantum confinements and photoluminescence properties [34]. The strong fluorescence of the GQDs makes them useful in cellular imaging. Owing to graphene family materials’ excellent properties, they possess huge potential for drug/gene delivery, biological sensing/imaging applications, and tissue engineering [35–39]. However, challenges still exist as to the long-term bio-safety and capability to induce cells osteogenic differentiation of graphene family materials. Here we review comprehensively recent progress and achievements in graphene and its derivatives. Simultaneously, we critically analyze the in vitro and in vivo bio-safety and discuss the feasibility of various biomedical applications of graphene family materials for bone tissue regeneration.

## Challenges in Determining the Bio-Safety of Graphene Family Materials

### Challenges in Determining the In Vitro Bio-Safety

Before graphene family materials are considered for clinical trial, they should be evaluated rigorously by its cytotoxicity and biocompatibility [38]. “Is graphene a biocompatible material?” The answer is still controversial. The raw graphene without any functionalization is hydrophobic and easily agglomerates in aqueous media [34, 40]. On hydrophobic surfaces, a dense layer of nonspecific proteins can displace water from the surface and immediately accumulate on the materials, resulting in an immunological recognition of the nanoparticles [41]. Thus, chemical functionalization, including oxidation, reduction, and introduction of functional groups, is a prerequisite for graphene used in biomedical applications, which increase the hydrophilicity of graphene. Graphene family materials with different functionalities, having different chemical properties, exert different toxicities [13]. Soumen et al. found that rGO was less toxic than GO. It was interesting to see that oxidative stress boosted with an increasing extent of oxygen functional group density on the rGO surface. They concluded that the functional group density on the GO sheet was one of the key factors in mediating cellular cytotoxicity [31]. Apart from surface functionalization, the cytotoxicity of graphene family materials was influenced by numerous factors, including their concentration, size, and shape [42].

Firstly, some researches demonstrated that graphene family materials had dose-dependent cytotoxicity with or without time-dependent cytotoxicity. For instance, Chang et al. reported that a slight loss of cell viability was observed at high concentration of GO (≥ 50 μg/mL) and GO can induce intracellular accumulation and cause a dose-dependent oxidative stress in a lung carcinoma epithelial cell line (A549) [43]. Wei et al. demonstrated that pristine GO inhibited the proliferation of bone mesenchymal stem cells (BMSCs) at a high concentration of 10 μg/mL, while enhanced proliferation of BMSCs at a low concentration of 0.1 μg/mL [44]. Similarly, a decreased number of cells was observed clearly with 200 μg/mL of GO and a greater cytotoxicity effect was reported with 300 μg/mL of GO [45]. What’s more, Kim et al. found that preosteoblasts (MC3T3-E1) viabilities were slightly affected by rGO at concentrations < 62.5 μg/mL, but were significantly (*p* < 0.05) diminished at higher concentrations (≥ 100 μg/mL) [23]. In addition, CXYG, GQDs both showed little cytotoxic potential when applied at low concentrations [34, 46]. Put simply, graphene family materials are cytocompatible at low concentration with little negative influence on cell morphology, viability, and proliferation, but the concentration is not the single pertinent factor.

Secondly, it is indicated that the diverse shapes, such as layer, nanosheets and flakes, ribbons, and dots, also contribute to the complexity of graphene family’s cytotoxicity [40]. Talukdar et al. evaluated the cytotoxicity of graphene nano-onions (GNOs), GO nanoribbons (GONRs), and GO nanoplatelets (GONPs). The CD_50_ values followed the trend GNOs > GONRs > GONPs, indicating that GONRs were more cytotoxic compared to GONPs [47]. Thus, the shape of the graphene family nanomaterials is also a key component in mediating cytotoxicity. For instance, graphene and multi-walled carbon nanotubes (MWNTs) have different shapes (flat atomic sheets for graphene and tubular for nanotubes), but their chemical composition and crystalline structure are similar. GO did not show the cell growth inhibitory activity of SK-N-SH cells until at 50 μg/mL. In comparison, MWCNTs inhibited the proliferation of the cell at low concentration (6.7 μg/mL), indicating its acute cytotoxicity. For HeLa cells, GO exhibited minor growth inhibitory activity even at concentration up to 50 μg/mL, whereas MWCNTs had moderate cytotoxicity on HeLa cells [48]. They depended this phenomenon on their different shape and varied physical/chemical manners. Graphene family materials were expected to have minor interaction with the cellular membranes because of the flat shapes. Tubular shape of MWCNTs promoted penetration of membranes, resulting in the cytotoxicity [48–50]. Another important information is that the cytotoxicity of nanostructured graphene derivatives is also cell-type dependent besides the dependence of functionalization, concentration, size, and shape. As a neural cell line, SK-N-SH cells exhibited more sensitivity than HeLa cells to the adverse effects of nanostructured graphene derivatives [48].

Thirdly, size also plays an important role on bio-safety of graphene family materials. Yoon et al. evaluated that the size-dependent cytotoxic effect of graphene nanoflakes via a cell-based electrochemical impedance biosensor. They found that the smaller graphene nanoflakes (30.9 ± 5.4 nm) induced apoptosis because of higher uptake by cells while the larger graphene nanoflakes (80.9 ± 5.5 nm) which mostly aggregated on cell membranes caused less toxicity [51]. It is well known that cellular uptake properties of nanomaterials may influence cell proliferation, differentiation, and nanoparticle excretion [52]. Mu et al. elaborated the likely size-dependent uptake mechanisms of protein-coated GO nanosheets and observed that larger nanosheets (860 ± 370 nm) first attached onto cell surface followed by membrane invagination, extending of pseudopodia and finally entered cells mainly through phagocytosis, while smaller nanosheets (420 ± 260 nm) entered cells predominantly through clathrin-mediated endocytosis [33]. Das et al. seed human umbilical vein endothelial cells (HUVEC) in 10 μg/mL of GO and rGO with different size sheets (800 nm and 400 nm). The results showed that the smaller sized sheets were more toxic than larger ones in the MTT assay. And then, the larger-sized GO and rGO (800 nm) were ultrasonicated in order to be broken into smaller sizes (70 nm). Increased cytotoxicity was observed after ultrasonication, indicating that the smaller sized GO and rGO exhibited more toxicity [31]. Similarly, MCF7 cells were exposed to four sized samples of GO (744 ± 178 nm, 323 ± 50 nm, 201 ± 28 nm, and 100 ± 10 nm). Compared to the untreated cells, no cytotoxicity was observed in vitro even after 72-h exposure to the larger-sized GO dispersions (744 ± 178 nm) while the treatment with 100 ± 10 nm-sized GO dispersions resulted in a decrease in cell proliferation to approximately 50% of untreated cells [53]. From the results above, a wide range of sizes of graphene family materials were researched, from 30 to 860 nm. And we seem to get the conclusion that smaller-sized graphene family materials are more toxic than larger-sized ones. But a different team has a different standard to define the size scale of graphene and its derivatives. Thus, this conclusion maybe debatable. Meanwhile, it was reported that nano-sized graphene family materials were much safer for biomedical applications [54]. Size-control synthesis of graphene family materials needs to be considered prudently in subsequent researches.

It is concluded that the cytotoxicity of graphene is critically related to the variety of graphene family, chemical functionalization, concentration, shape, and size. In the future, we aim to fabricate biocompatible devices with better interactions with cells, tissues, or organisms by better control of concentration and size, by modifying the graphene family with various types of functional groups.

### Challenges in Determining the Bio-safety and Biodistribution In Vivo

**Fig. 1:**
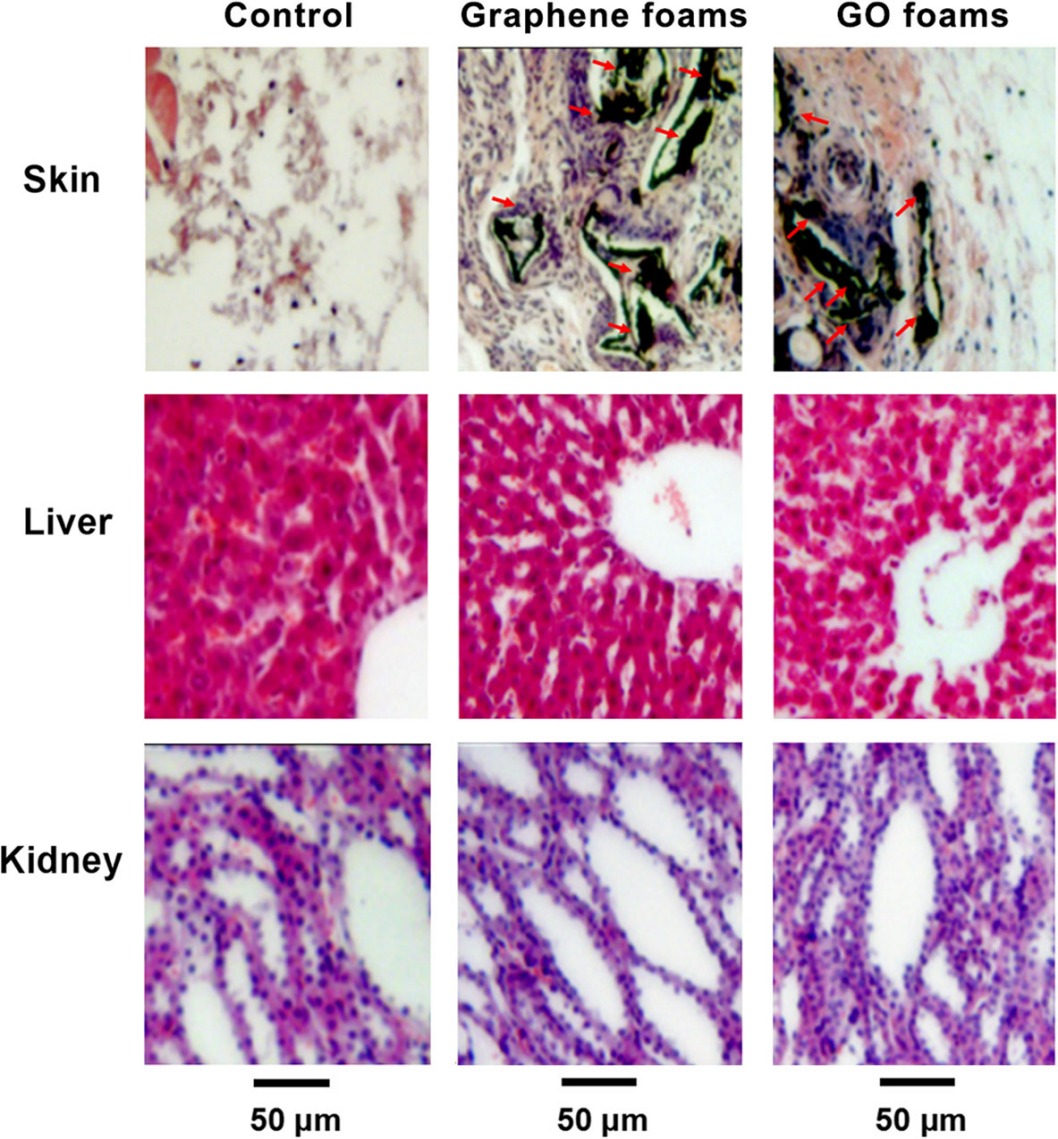
Representative HE-stained images of major organs (implantation region, liver, and kidney collected from the rats) implanted with graphene foams, GO foams, or nothing at day 14 post-implantation. No obvious organ damage or lesion was observed. Reproduced from ref. [40] with permission from the Journal of Nanoparticle Research

In order to further detect whether graphene family materials are biocompatible materials and to enhance the proposed use in widespread applications, in vivo experiment is an indispensable method. A lot of researches about biocompatibility and biodistribution of graphene family materials in vivo are nearly consistent with their cellular studies. Chowdhury et al. applied zebrafish embryo to larger-sized GO dispersions and found no increased mortality of embryo compared with the control group, while reduced embryo viability was observed in the smaller-sized GO dispersions [53]. GO did not lead to significant increase of apoptosis in embryo while MWCNTs resulted in serious morphological defects in developing embryos even at relatively low concentration of 25 mg/L [48]. These studies further indicated that the in vivo toxicity greatly lies upon the sizes, concentrations, and shapes of graphene and its derivatives. Moreover, graphene family materials are usually exposed to animal models through intravenous injection, inhalation, or subcutaneous implantation. Thus, changes in toxicity, general histology, and biodistribution are varying. Li et al. evaluated the toxicology of nanoscale GO in mice via intravenous injection and found that GO was mostly retained in the liver, lung, and spleen and induced damage, chronic hepatitis, and lung fibrosis. A polyethylene glycol (PEG) coating of GO (GO-PEG) could reduce the retention of GO in the liver, lung, and spleen and alleviate the acute tissue injuries [55]. Duch et al. explored strategies to reduce the toxic effect of graphene nanomaterials in the lung because they found that GO had higher toxicity than aggregated graphene and Pluronic-dispersed graphene when administered directly to the lungs of mice, inducing severe and persistent lung injury. The toxicity was significantly moderated by the fabrication of pristine graphene through liquid phase exfoliation and was further minimized when dispersed with the block copolymer Pluronic [56]. Zha et al. identified the short-term (first 2 weeks post-implantation) and long-term (7 months) in vivo toxicity and performance of 3D graphene foams (GFs) or graphene oxide foams (GOFs) in a rat model of subcutaneous implantation. The blood analysis showed that GFs and GOFs did not induce appreciable hematologic, hepatic, or renal toxicity after implantation and no significant degradation was observed after at least 7 months implantation. Only granulomas existed for a long time in the implantation site was observed. HE stained images showed better in vivo biocompatibility (Fig. 1) [40]. The reason why Zha et al. attained more positive results than other studies as aforementioned probably is the different routes of administration. Subcutaneous experiment was the very direct and effective way to assess the in vivo biocompatibility of implanted materials [57], which may exert an effect on the contact patterns, deposit locations, even the degradation pathways of the graphene family nanomaterials in vivo [58]. Controlling the degradation of composites is of vital importance in tissue engineering,

Generally, cellular researches are outstanding for a preliminary cytotoxicity analysis, understanding of the likely mechanism of interaction with cells. But it is a much more complicated microenvironment in vivo. Understanding how graphene family materials behave in humid corrosive microenvironments is also pivotal. The biocompatibility of graphene family materials is nontrivially related to the concentration, the varieties of functional groups, types of graphene family, sizes, and shapes. But the mechanism still needs to be further investigated in detail and thoroughly. However, the assessment of in vivo bio-safety is comparably not much, especially the long-term biocompatibility and biodistribution, which need us to pay more attention. Although some papers raise concerns about bio-safety, the potential versatility that graphene family uniquely offers have made it a competitive candidate of option for biomedical applications.

## Antibacterial Activity of Graphene Family Materials

Bone remolding and new bone formation cannot be completely successful without a sterile microenvironment of the bone defect. In fact, the treatment of infective bone defect is still a major challenge [59]. Because of the large bone defect and infective problem, the treatment is hard and patients need a long-term period of recovery. Thus, the capability of bacteria inhibition of graphene family materials helps a lot. Graphene family materials are believed to possess the capability of antibacteria (Table 1). Liu et al. proposed a three-step antimicrobial mechanism, including (1) initial cell deposition on graphene-based materials, (2) membrane stress caused by direct contact with sharp nanosheets, and (3) the ensuing superoxide anion-independent oxidation [60]. However, Mangadlao et al. thought that the surface of graphene was primarily responsible for antimicrobial activity and not the edges. When in contact with bacteria, graphene served as an electron acceptor that pumped the electron away from the bacterium’s membrane creating an independent oxidative stress [61]. Meanwhile, Li et al. provided new insights for the better understanding of antibacterial actions of graphene film. They hold the opinions that the antibacterial activity of graphene family materials did not stem from reactive oxygen species (ROS)-mediated damage, but via electron transfer interaction from microbial membrane to graphene [62], whereas Panda et al. proved that a synergetic influence of nonoxidative electron transfer mechanism and consequent ROS-mediated oxidative stress to the bacteria induced an enhanced antimicrobial activity of naturally derived GO-metal films [63].

**Table 1:** Antibacterial activity of graphene family materials

Graphene family materials	Substrate or other molecules	Fabrication methods	Bacteria	Antimicrobial outcomes	Ref.
GO and rGO		Vacuum filtration	*E*. *coli* (G-)	rGO possessed antibacterial properties that were only slightly lower than those of GO, while their cytotoxicity was significantly higher than GO’s.	[153]
rGO and GO		Electrophoretic deposition method	*E*. *coli* (G-) *S*. *aureus* (G+)	1. *E*. *coli* bacteria with an outer membrane were more resistant to the cell membrane damage caused by the GO and rGO than *S*. *aureus* lacking the outer membrane. 2. The rGO exhibited stronger toxicity against two bacteria than the GO.	[154]
Graphite, graphite oxide, GO, rGO			*E*. *coli* (G-)	1. Antibacterial activity: GO > rGO > graphite > graphite oxide	[60]
Monolayer graphene film	Conductor Cu semiconductor Ge insulator SiO2	Chemical vapor deposition (CVD)	*E*. *coli* (G-) *S*. *aureus* (G+)	1. Graphene@Cu and Graphene@Ge can surprisingly inhibit the growth of both bacteria, especially the former. 2. The proliferation of both bacteria cannot be significantly restricted by the graphene film on SiO2.	[62]
Bare GO sheets		Modified Hummers’ method	*E*. *coli* (G-) *B*. *subtilis* (G+)	1. Bare GO sheets indeed kill bacteria. 2. Masking GO sheets basal planes via noncovalent adsorption resulted in GO inactive against bacteria.	[155]
GO	Polyethylene terephthalate (PET)	Langmui–Blodgett (LB) technique	*E*. *coli* (G-)	1. Antibacterial activity was layer dependent. 2. Contacting with the edges was not a fundamental part of GO’s antimicrobial mechanism.	[61]
GO			*E*. *coli* (G-)	The smaller-sized GO sheet increased the antimicrobial activity of the material.	[156]
rGO	l-cysteine (Cys) modified Ag	Mix	*E*. *coli* (G-) *S*. *aureus* (G+)	This nanocomposite showed excellent electrocatalytic activity against glucose and bactericidal property against *E*. *coli* and *S*. *aureus* (MIC:0.3 mg/ml and MBC:0.6 mg/ml).	[157]
Multi-layer-numbers GO	Pure titanium plates	Colloidal dispersion	*E*. *coli* (G-) *S*. *aureus* (G+)	Increasing the layer-number of graphene oxide resulted in the augment of ROS levels and the wrinkling, which led to the bacteria inhibition.	[118]
GO sheets; rGO sheets	Titanium foil	Evaporation-assisted electrostatic assembly and one-pot assembly	*S*. *aureus* (G+)	Both types of layer showed good antibacterial activity whereby around 50% anti-adhesion effects and considerable anti-biofilm activities were observed.	[120]
GO-Ag nanohybrid	Bacterial cellulose (BC)	GO-Ag nanohybrid synthesis via Response Surface Methodology	*E*. *coli* (G-) *S*. *aureus* (G+)	1. GO-Ag nanohybrid exhibited synergistically strong antibacterial activities at rather low dose. 2. GO-Ag nanohybrid is more toxic to *E*. *coli* than that to *S*. *aureus*.	[158]
GO	Polydopamine (PDA) modified porous Ti scaffolds	A new drug delivery system (BMP-2; vancomycin (Van))	*S*. *epidermidis* (G+)	GO/Ti scaffold encapsulated with Van inhibited the proliferation of *S*. *epidermidis* to a large extent, compared to that of scaffolds without Van.	[149]
GO	Silicone rubber sheets	The activated sheets were immersed into the GO dispersion.	*E*. *coli* (G-) *S*. *aureus* (G+)	The GO coatings caused a significant viability loss up to 85.8% for *E*. *coli* and 72.4% for *S*. *aureus*, showing stronger antibacterial activity against *E*. *coli* bacteria than their activities against *S*. *aureus* bacteria.	[159]
GO	Metallic films, such as Zn, Ni, Sn, and steel		*E*. *coli* (G-)	It is also found that such activities are directly correlated to the electrical conductivity of the GO-metal systems; the higher the conductivity the better is the antibacterial activity.	[63]

Although it remains uncertain how the physicochemical properties of graphene-based sheets influence their antimicrobial activity, the ability of antibacteria of graphene family material is worthy us studying and taking further advantage of.

## Graphene Family Materials Mediate Cells into Osteogenic Differentiation and Promote Bone Regeneration In Vivo

**Fig. 2:**
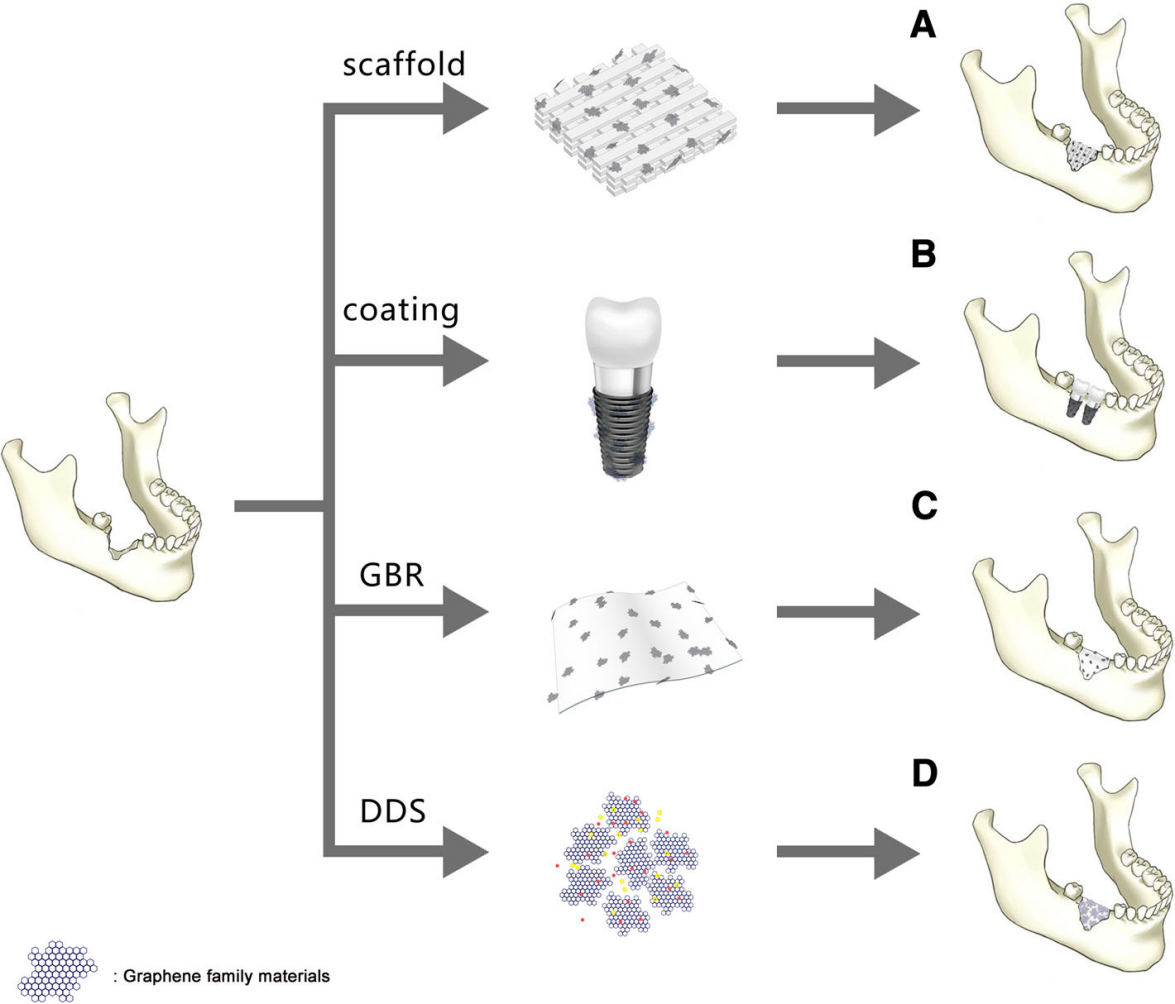
A. Graphene family materials as scaffold or a reinforcement material in scaffold for bone regeneration. B. Graphene family materials as coating transferred onto the substrate for bone regeneration. C. Graphene family as an additive in guided bone membrane. D. Graphene family materials as drug delivery system facilitates bone regeneration

Many scholars have pointed out that graphene not only can allow the attachment and proliferation of cells (e.g., dental pulp stem cells [64, 65], bone marrow stem cells [8, 20, 66, 67], periodontal ligament stem cells [68], human osteoblasts [69], fibroblast cells [70], tumor cells [43]) without signs of apparent cytotoxicity but also can induce early cells osteoblastic differentiation and yield high degrees of mineralization [20, 64–68]. At present, numerous teams painstakingly did plentiful studies to design new strategies of applying graphene family nanomaterials as a scaffold or an additive to the scaffold, as a coating onto the substrate material surface, as a guide bone regeneration membrane, and as a drug delivery vehicle (Fig. 2). They tried to use graphene family materials to improve the certain properties of the substrate material even further and confer a bioactive character to the substrate-based composites.

### Graphene Family Materials as Scaffold or a Reinforcement Material in Scaffold

The most common strategy for bone tissue engineering is simulating the natural process of bone remolding and regeneration. The strategy can be satisfied by three dimensional (3D) biocompatible, biodegradable, and osteoconductive or osteoinductive scaffold [3]. This kind of scaffold can offer an ideal microenvironment to mimic the extracellular matrix (ECM) for osteogenic cells attachment, migration, proliferation, and differentiation as well as for the carriers of growth factors [6]. Graphene as a promising biocompatible scaffold can make the big surface area available for cell spreading and even osteogenic differentiation in the substrate [20]. For example, 3D graphene foams employed as culture substrates for human mesenchymal stem cells (hMSC) provided evidence that they were able to maintain stem cell viability and promote osteogenic differentiation [66]. Moreover, 3D graphene (3DGp) scaffold as well as 2D graphene (2DGp) coating was proved to be able to induce the differentiation of periodontal ligament stem cells (PDLSC) into mature osteoblasts by the higher levels of mineralization and upregulated bone-related gene and proteins on graphene, with or without the use of chemical inductors [68].

Nowadays, the diverse biomaterials serving as scaffolds spring up like mushrooms. The potentially suitable synthetic scaffolds for use in bone regeneration includes calcium phosphate, such as hydroxyapatite (HA) [71]; β-tricalcium phosphate (β-TCP) [72]; synthetic or bio-polymers, such as poly-lactic acid (PLA) [73], poly-glycolic acid (PLGA) [74], polycaprolactone (PCL) [75], chitosan (CS) [1], and collagen [76]; and composites of the upper mentioned materials [77, 78]. But now, one of the most important concerns is the mechanical properties of scaffolds. Since natural bone exhibits super-elastic biomechanical properties with a Young’s modulus value in the range of 7–27 GPa [79], the ideal scaffolds should mimic strength, stiffness and mechanical behavior of natural bone. Graphene family materials can be added as a reinforced material in scaffolds aiming to strengthen mechanical properties and improve physicochemical characterization. For example, the pure PCL scaffold had a tensile strength of 1.61 MPa, an elongation of 122%, and a Young’s modulus of 7.01 MPa. The addition of GO (2%) resulted in a considerable increase in tensile strength to 3.50 MPa, elongation to 131%, and Young’s modulus to 15.15 MPa [80].

Stimulated by the success of using graphene family materials as a reinforcement material, many teams combined biocompatibility provided by synthetic or bio-polymers with remarkable physical properties of graphene family materials. They expected to attain an ideal composite scaffold with improved mechanical properties, suitable porosity, structural designs, and excellent biocompatibility, to support and induce new bone formation.

#### Graphene Family with Calcium Phosphate-Based Materials

Human bone consists of 30% organic matter, mostly collagen, and 70% inorganic matter, mostly hydroxyapatite (HA; Ca_10_(PO_4_)_6_(OH)_2_) [81, 82]. Synthetic calcium phosphate-based materials such as HA, β-tricalcium phosphate (β-TCP), and calcium phosphate cements (CPC) are popular scaffolding materials because of their similar compositions and structures as natural mineral phase of bone and their good bone-forming abilities [83–85]. In particular, because of the good osteoconduction and osteoinduction ability of HA [86], it has been widely used for a long time as artificial bone grafts in orthopedic or maxillofacial surgery to repair bone defect areas [11, 71]. However, the inherent drawbacks of HA material should be improved, such us shaping difficulty, peculiar brittleness, and low fracture toughness [87, 88]. It was reported that graphene family material-reinforced HA composites were developed and significantly enhanced fracture toughness and biological performances. For instance, HA/graphene composites were prepared by spark plasma sintering (SPS), which endowed HA acceptable strength [89]. Raucci et al. combined HA with GO in two different approaches: in situ sol–gel approach and biomimetic approach. The HA–GO obtained by in situ sol–gel approach enhanced the cell viability of hMSCs and induced osteoblastic differentiation without using osteogenic factors. The HA–GO formed via biomimetic approach sustained cell viability and proliferation [90]. Moreover, the reduced graphene oxide (rGO) can be also used as reinforcement material for HA. The fracture toughness of the HA–rGO composites reached 3.94 MPa m^1/2^, a 203% increase compared to pure HA. The HA–rGO enhanced the cell proliferation and osteoblastic differentiation, which was assessed by alkaline phosphatase (ALP) activity of the human osteoblast cells [91]. In addition, Nie et al. successfully synthesized rGO and nano-hydroxyapatite (nHA) 3D porous composites scaffold (nHA@rGO) via self-assembly. The GO solution blended with nHA water suspension which was heated to induce the self-assembly process. At last, the reaction products were freeze-dried to obtain the 3D porous scaffold. The nHA@rGO scaffold can significantly facilitate the cell proliferation, ALP activity, and osteogenic gene expression of rat bone mesenchymal stem cells (rBMSCs). And in vivo experiment elucidated that 20% nHA-incorporated rGO (nHA@rGO) porous scaffold can accelerate healing the circular calvarial defects in rabbits [92]. Besides, not only double components but also tricomponent had excellent performances with good cytocompatibility and improved hydrophilic and mechanical properties [93–95].

**Fig. 3:**
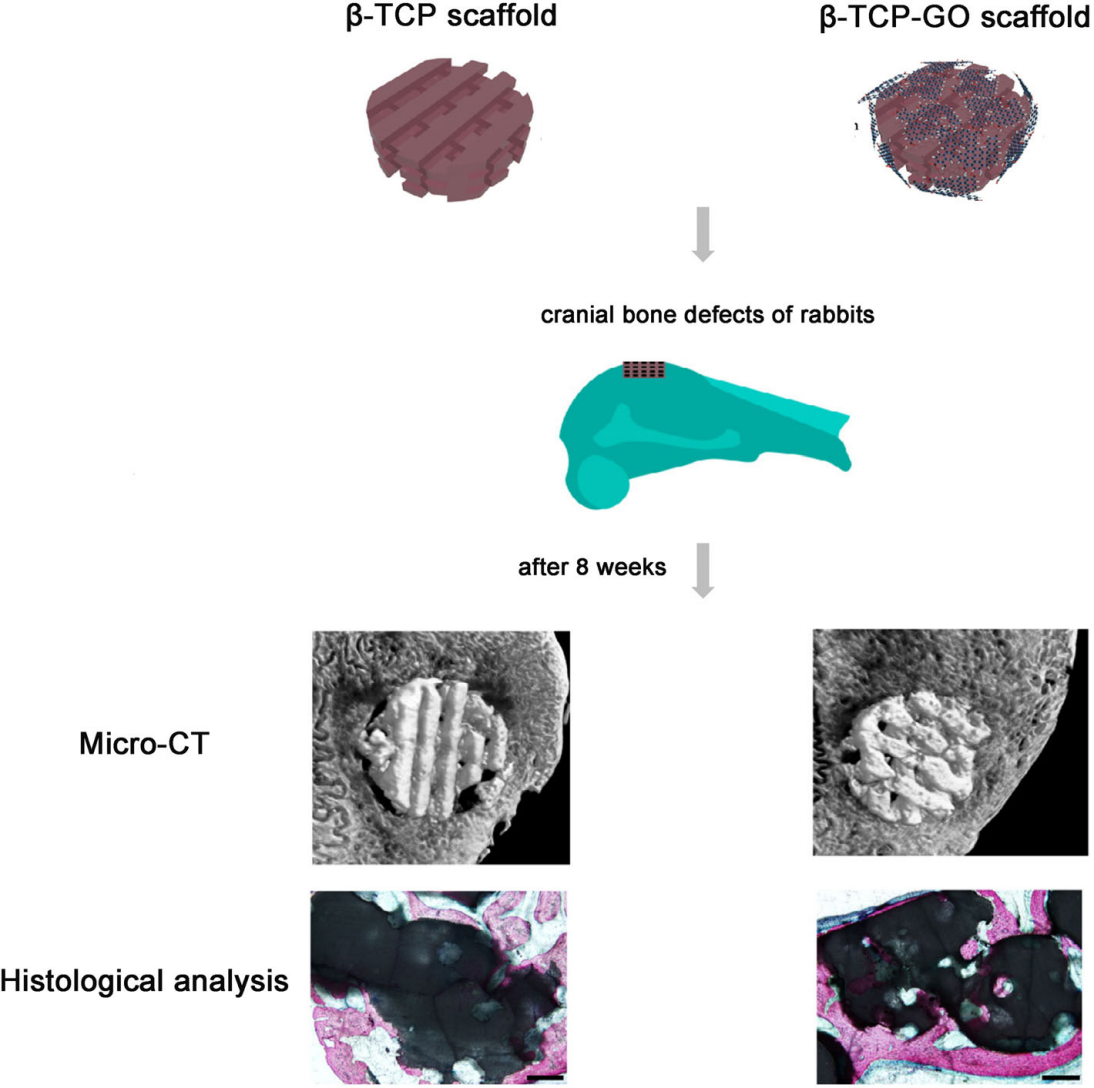
Scheme illustration for β-TCP and β-TCP-GO scaffolds stimulated the in vivo osteogenesis. Micro-CT analysis and histological analysis of in vivo bone formation ability for the β-TCP and β-TCP-GO scaffolds after implanted in the cranial bone defects of rabbits for 8 weeks. Reproduced from ref. [85] with permission from the Journal of Carbon

Tricalcium phosphate, analog of calcium phosphate, is a tertiary calcium phosphate also known as bone ash [Ca_3_(PO_4_)_2_]. It serves as an abundant origin for calcium and phosphorus, which can be easily absorbed. Beta-tricalcium phosphate (β-TCP) is highly biocompatible and creates a resorbable interlocking network within the defect site to promote healing [96]. Wu et al. successfully synthesized 2D β-TCP-GO disks and 3D β-TCP-GO scaffolds. Compared to β-TCP and blank control, the 2D β-TCP-GO disks significantly enhanced the proliferation, ALP activity, and osteogenic gene expression of hBMSCs by activating the Wnt-related signaling pathway, indicating the excellent in vitro osteostimulation property of GO-modified β-TCP [85]. It is known that the Wnt canonical signaling pathway plays a nontrivial role in regulating cellular activities such as cell proliferation, differentiation, and morphogenesis [97, 98]. In vivo study exhibited that 3D β-TCP-GO scaffolds had greater new bone formation in the calvarial defects than pure TCP scaffold (Fig. 3) [85]. A novel scaffold, calcium phosphate cement incorporated GO-Cu nanocomposites scaffolds (CPC/GO-Cu) facilitated the adhesion and osteogenic differentiation of rBMSCs, which were confirmed that they can upregulate the expression of Hif-1α in rBMSCs by activating the Erk1/2 signaling pathway and induced the secretion of vascular endothelial growth factor (VEGF) and BMP-2 protein. Furthermore, the CPC/GO-Cu scaffolds were transplanted into rat with critical-sized calvarial defects and the results showed that the scaffolds (CPC/GO-Cu) significantly promoted angiogenesis and osteogenesis in the defect areas [99].

#### Graphene Family with Chitosan

**Fig. 4:**
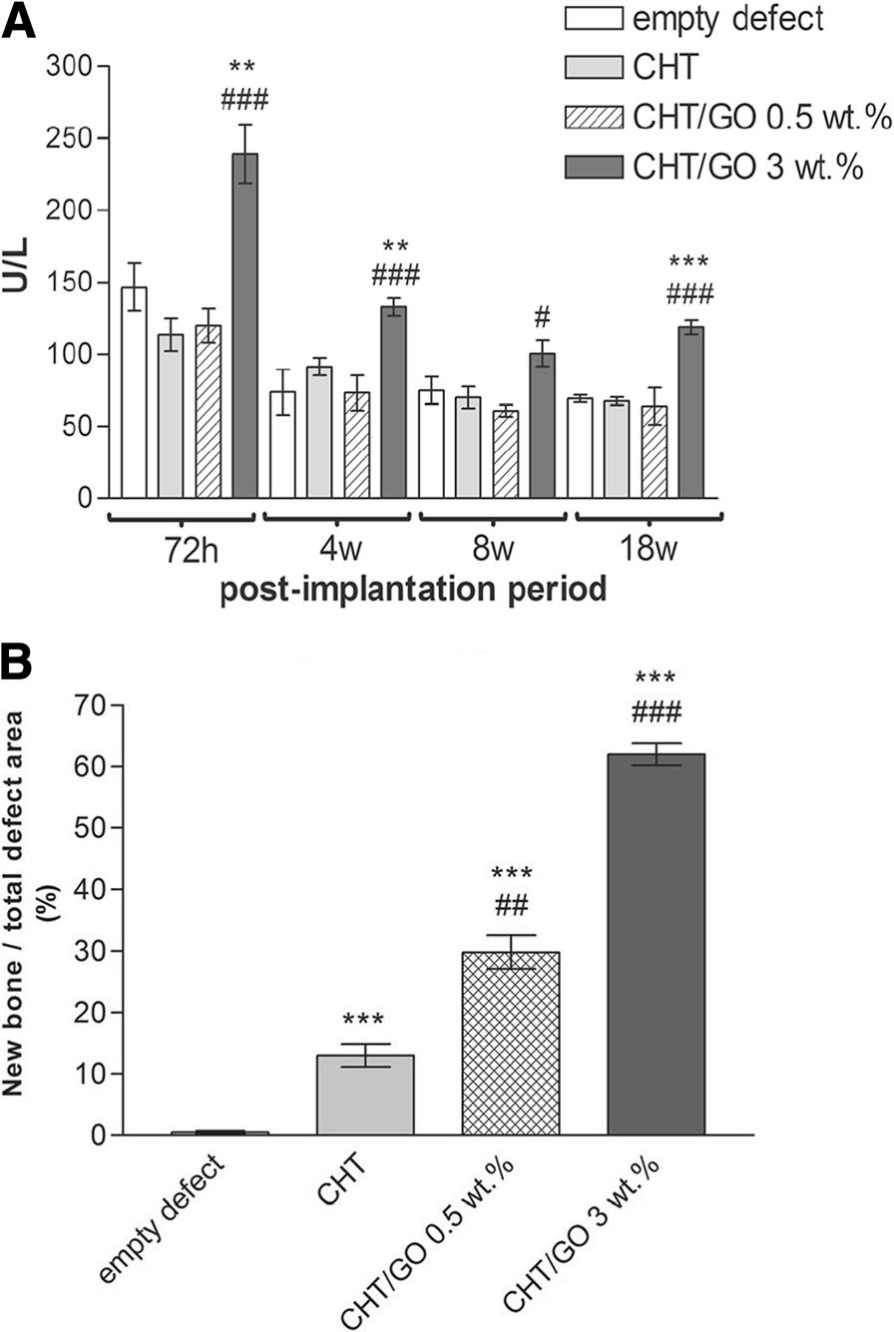
**a** ALP activity in mice calvaria defects implanted with CHT/GO and **b** histomophometric analysis of Masson Goldner trichrome-stained sections. ###*p* < 0.001 vs CHT; **p < 0.01 vs control; ***p < 0.001 vs control. Reproduced from ref. [3] with permission from the Journal of Scientific Reports

Chitosan (CS), a highly versatile biopolymer, derived from the shells of crustaceans [1, 87], has a hydrophilic surface that promotes cell adhesion and proliferation and its degradation products are nontoxic. Chitosan is biocompatible, osteoconductive, hemostatic, and can be easily converted into the desired shapes [2]. Besides, chitosan can promote bone matrix of mineralization [1] and minimize the inflammatory response after implantation [100]. All properties above make chitosan especially attractive as a bone scaffold material. But the most challenging part is the obtainment of CS-based scaffolds with good mechanical properties and processability [101]. Interestingly, CS/GO scaffolds have high water-retention ability, porosity, and hydrophilic nature [101, 102]. The CS-based 3D materials were enriched with GO in different proportions (0.5 wt% and 3 wt%). The new developed CS/GO 3 wt% scaffold was expected to be ideally designed for bone tissue engineering applications in terms of biocompatibility and properties to promote cell growth and proliferation [103]. Another CHT/GO scaffold with 0, 0.5, and 3 wt.% GO were prepared by freeze-drying method. Similarly, the CS/GO 3 wt% scaffolds significantly enhanced the ALP activity in vitro and the new bone formation in vivo, suggesting a positive contribution of 3 wt% GO to the efficiency of osteogenic differentiation process (Fig. 4) [3]. All results proved that CS/GO scaffolds could be a feasible tool for the regeneration of bone defects, and the addition of a 3 wt% of GO to material composition could have a better impact on cell osteogenic differentiation.

**Fig. 5:**
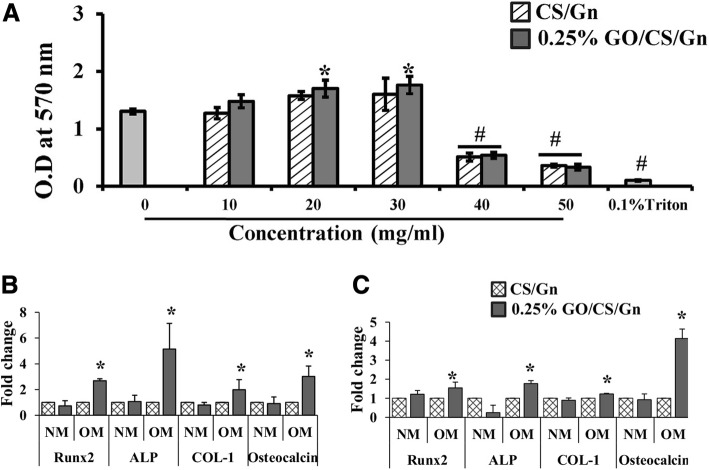
**a** MTT assay after incubation of CS/Gn scaffolds and 0.25% GO/CS/Gn scaffolds with media for 48 h. The asterisk indicates a significant increase versus control, and the pound sign indicates a significant decrease versus control (*p* < 0.05). **b**, **c** Expression of osteogenic-related genes (RUNX2, ALP, COL-1, and OC) in mMSCs cultured on CS/Gn scaffolds and 0.25% GO/CS/Gn scaffolds for 7 and 14 days measured by quantitative RT-PCR. Reproduced from ref. [104] with permission from the Journal of International Journal of Biological Macromolecules

Moreover, some tricomponent composites, such as CS, GO, and HA can release more Ca and P ions compared to the pure HA nanoparticles, displaying a high bioactivity of the composite scaffold [87]. Ravichandran et al. fabricated a unique composite scaffold, GO–CS–HA scaffold, and the incorporation of GO enhanced the tensile strength of CS up to 8.2 MPa and CS–HA to 10 MPa. And the results demonstrated that GO–CS–HA scaffolds facilitated cell adhesion and proliferation, meanwhile showed improved osteogenesis in in vitro tests [2]. Another tricomponent composite scaffold, containing CS, gelatin (Gn), and different concentrates of graphene oxide (0.1%, 0.25%, 0.5%, and 1% (*w*/*v*) GO) showed better physic-chemical properties than CS/Gn scaffolds. The addition of GO at the concentration of 0.25% to CS/Gn scaffolds exhibited enhanced absorption of proteins, extensive apatite deposition. The 0.25% GO/CS/Gn scaffolds were cyto-friendly to rat osteoprogenitor cells, and they enhanced differentiation of mouse mesenchymal stem cells into osteoblasts in vitro (Fig. 5). The tibial bone defect filled with 0.25% GO/CS/Gn scaffolds showed the growth of new bone and bridging the defect area, indicating their biocompatible and osteogenic nature [104]. Thus, no matter bicomponent or tricomponent composites scaffolds, the addition of graphene family materials to chitosan can favorably improve the mechanical properties and regulate the biological response of osteoblasts, promoting osteogenic differentiation.

#### Graphene Family with Other Synthetic or Bio-polymers

Sponge scaffolds of type I collagen, the major organic component of bone [81], have been clinically applied as scaffolds to regenerate bone tissue [105, 106]. Because collagen scaffolds (elastic moduli:14.6 ± 2.8 kPa) are relatively soft, the combination with GO is expected to enhance the elastic modulus of collagen scaffolds and to improve the osteogenic differentiation of MSCs for bone regeneration. The covalent conjugation of GO flakes to 3D collagen scaffolds (elastic moduli: 38.7 ± 2.8 kPa) increased the scaffold stiffness by threefold and did not negatively affect the viability of BMSCs. The enhanced osteogenic differentiation observed on the stiffer scaffolds were likely mediated by BMSCs mechanosensing because the molecules involved in cell adhesion to stiff substrates were either upregulated or activated [107]. Moreover, the development of new biomaterials utilizing graphene family materials with high osteogenic capacity is urgently pursued (Table 2).

**Table 2:** The scaffolds made of graphene family materials and other synthetic or bio-polymers

Graphene family materials	Synthetic or bio-polymers	Fabrication methods	The improvement of physical or mechanical properties	Key results of experiments in vitro	Key results of experiments in vivo	Ref.
GO	PCL	Electrospinning process	Highly porous nature; an increase in tensile strength, elongation and Young’s modulus	Better biological characteristics with high cell viability		[80]
rGO	Macro–mesoporous bioactive glass (MBG); osteoblast-specific aptamer (AP)	Sol–gel method	Macroporous structure with fully interconnected open pores; excellent mechanical properties with a Young’s modulus of ~ 80 kPa	Accelerated the osteogenic differentiation of rat osteoblasts by up-regulating the mRNA expression level of four osteoblast markers sinificantly.	In the large bone defects of the rat femurs, the new bone appeared both peripherally and centrally in rGO-MBG-AP scaffold.	[160]
rGO	Polypyrrole (PPY); casein phosphopeptide (CPP)	Electrostatic self-assembly method	Excellent hydrophilic property and water uptake performance	Promoted the rapid formation of hydroxyapatite in the biomimetic mineralization; enhanced the adhesion, proliferation and osteogenic differentiation of MC3T3-E1 cells.		[161]
rGO	PPY; HA	Electrostatic layer-bylayer assembly strategy; biomimetic mineralization	Better mechanical property with desired configuration, high specific surface area and large surface roughness.	Enhanced MC3T3-E1 cells adhesion and proliferation.		[162]
GO	Poly(3-hydroxybutyrate-co-4-hydroxybutyrate)	Electrospinning technique	Reduced the fiber diameter and enhanced porosity, hydrophilicity and mechanical properties of the scaffolds.	Improved cellular performance, and osteogenic differentiation in vitro.	Promoted osteogenesis and rapidly increased bone volume even at an early stage.	[163]
GO	Cellulose acetate (CA); nanofibrous	Electrospinning technique	Increased the Young’s modulus of the nanofibers in a GO dose-dependent manner	Facilitated adhesion and proliferation of BMSCs on the scaffolds; accelerated biomineralization; induced osteogenic differentiation of BMSCs		[164]
Graphene oxide carboxymethylation (cGO)	HA; silk fibroin (SF)	Biomimetic mineralization and simply mix	Higher compressive strength and compressive modulus, respectively	Stimulated BMSCs adhesion and proliferation, ALP secretion and mineral deposition more strongly		[165]
rGO	Zinc silicate (ZS); calcium silicate	Two-step spin-coating method	Increased annealing temperature	Suppressed the receptor activator of nuclear factor-κB-ligand-induced osteoclastic differentiation of mouse leukemic monocyte macrophages		[166]
rGO	PDMS	Dipped and dried	Good mechanical strength and with pore sizes ranging from 10 to 600 um	Accelerated growth and differentiation of human adipose stem cells to an osteogenic cell lineage		[167]
GO	Nano-HA; collagen; PLGA	Freeze-drying method	Improved the hydrophilicity and reinforced their mechanical strength; increased Young’s modulus (10.20 ± 1.28 GPa)	Enhanced cell adhesion and proliferation of MC3T3-E1		[168]
GO	Gelatin hydroxyapatite matrix	Freeze-drying method	Less brittleness	Induced osteogenic differentiation of human adipose derived mesenchymal stem cells without chemical inducer		[169]
Pristine graphene	PCL	3D printing	Increased hydrophilicity of the surface	Enhanced cell viability and proliferation		[170]
GO multi-walled carbon nanotube oxides (MWCNTO)	Poly (d, l-lactic acid) (PDLLA)	MWCNTO-GO was prepared via oxygen plasma etching (OPE)	High mechanical performance (~ 600 MPa)	Allowed for MG-63 cells interactions and the formation of mineralized matrix significantly facilitated osteoblast ALP activity	Superior influence on bone cell activity, promoting greater new bone formation	[171]

Up to now, these improved tricomponent systems for bone tissue engineering scaffolds possess good biocompatibility, which can promote cell attachment, proliferation, and have been reported mechanical properties matchable to those of natural bone. But the response to specific biological signals expressing, as well as the capabilities of enhancing cell differentiation and finally bone tissue regeneration, still needs to be explored further. Moreover, it has been reported that the pore structure (pore size, pore morphology, and pore orientation) and the elasticity of scaffolds were manipulated to regulate osteogenesis [108–110]. However, due to the complicated structure of porous and different elasticity accurately controlled of the scaffolds, it remains a major challenge to individually design specific pore architectures and elasticity 3D porous scaffolds that can stimulate bone regeneration. With the rapidly development of the science and technology, the emerging of the 3D-printing method may overcome this problem and open an avenue for bone tissue regeneration [85]. The in vitro bioactivity and excellent in vivo bone-forming ability of graphene family nanomaterials present a new prospect of developing a broad new type of multifunctional scaffolds for biomedical applications. Thus, we believe that the unraveled the molecular mechanisms behind will be revealed soon and graphene family materials still have attractive potential of applications in bone regeneration waiting us to explore.

### Graphene Family Materials as Coating

Graphene family materials have been widely applied in diverse forms of medical applications for bone regeneration. As a coating, graphene family materials can be transferred on two dimensional (2D) flat non-metal or metal substrates to induce spontaneous osteogenic differentiation of several types of mesenchymal stem cells (MSCs) [64]. Nayak et al. transferred graphene to four 2D non-metal substrates (polydimethylsiloxane (PDMS), polyethylene terephthalate (PET), glass slide, and silicon wafer with 300 nm SiO_2_ (Si/SiO_2_).) and investigated the influence of graphene on BMSCs differentiation. They summarized that the graphene coating was cytocompatible and contributed to enhance the osteogenic differentiation of BMSCs at a rate comparable to differentiation under the influence of BMP-2 in the osteogenic medium [20]. Similarly, Elkhenany et al. found that goat BMSCs, seeded on 2D graphene-coated plates underwent osteoblastic differentiation in culture medium without the addition of any specific growth factors [8]. Simultaneously, Lee et al. tried to explain the origin of how graphene coating could accelerate stem cell renewal and differentiation. They deemed that the strong noncovalent binding abilities of graphene allowed it to serve as a preconcentration platform for osteoblastic inducers, which facilitated BMSCs osteogenic differentiation [67]. The capability of graphene in modulating osteogenic differentiation is evident. How about its derivatives? GO coatings and rGO coatings all showed favorable cytocompatibility and enhanced spontaneous osteogenic differentiation by upregulating levels of ALP activity [111, 112].

Since titanium (Ti) and medical-grade Ti alloy have been extendedly applied in the orthopedic and dental fields [113–115], satisfactory osseointegration for titanium and its alloys is still a major challenge and need to be explored deeply in order to help the clinicians to promote the success or survive rate of implants and diminish the likely complications encountered after their placement [114, 116, 117]. Graphene family materials coated titanium and its alloys, serving as a new method to improve their capabilities of osseointegration at the tissue-implant interface, attracted widespread attention. For example, GO-coated titanium enhanced cell proliferation, upregulated levels of ALP activity and gene expression level of osteogenesis-related markers, and promoted the protein expression of BSP, Runx2, and OCN [117]. Qiu et al. made different thickness GO coatings on the pure titanium surfaces respectively by cathodal electrophoretic deposition. Interestingly, with the increasing thickness of GO, the ALP-positive areas improved, ECM mineralization increased [118]. Moreover, Zeng et al. firstly fabricated GO/HA composite coatings by electrochemical deposition technique on Ti substrate. The addition of GO facilitated both the crystallinity of deposited apatite particles and the bonding strength of the as-synthesized composite coatings [119]. It is well known that hydrophilic surface is biocompatible compared to hydrophobic surface. In the case of rGO coating, the rapid adsorption of serum protein improves hydrophilia of graphene surface and enhances cell adhesion. Jia et al. used evaporation-assisted electrostatic assembly and one-pot assembly to fabricate 2D GO-coated Ti and rGO-coated Ti, with tailored sheet size and surface properties. Compared to the contact angle of titanium (60.4°), the contact angle of GO-coated Ti and rGO-coated Ti were 20° and 14.2°, respectively, indicating the successful interfacial assembly of graphene and excellent wettability properties. The rGO-coated Ti elicited better cell adhesion and growth than bulk GO, while the latter evoked higher activity of osteogenic differentiation [120].

**Fig. 6:**
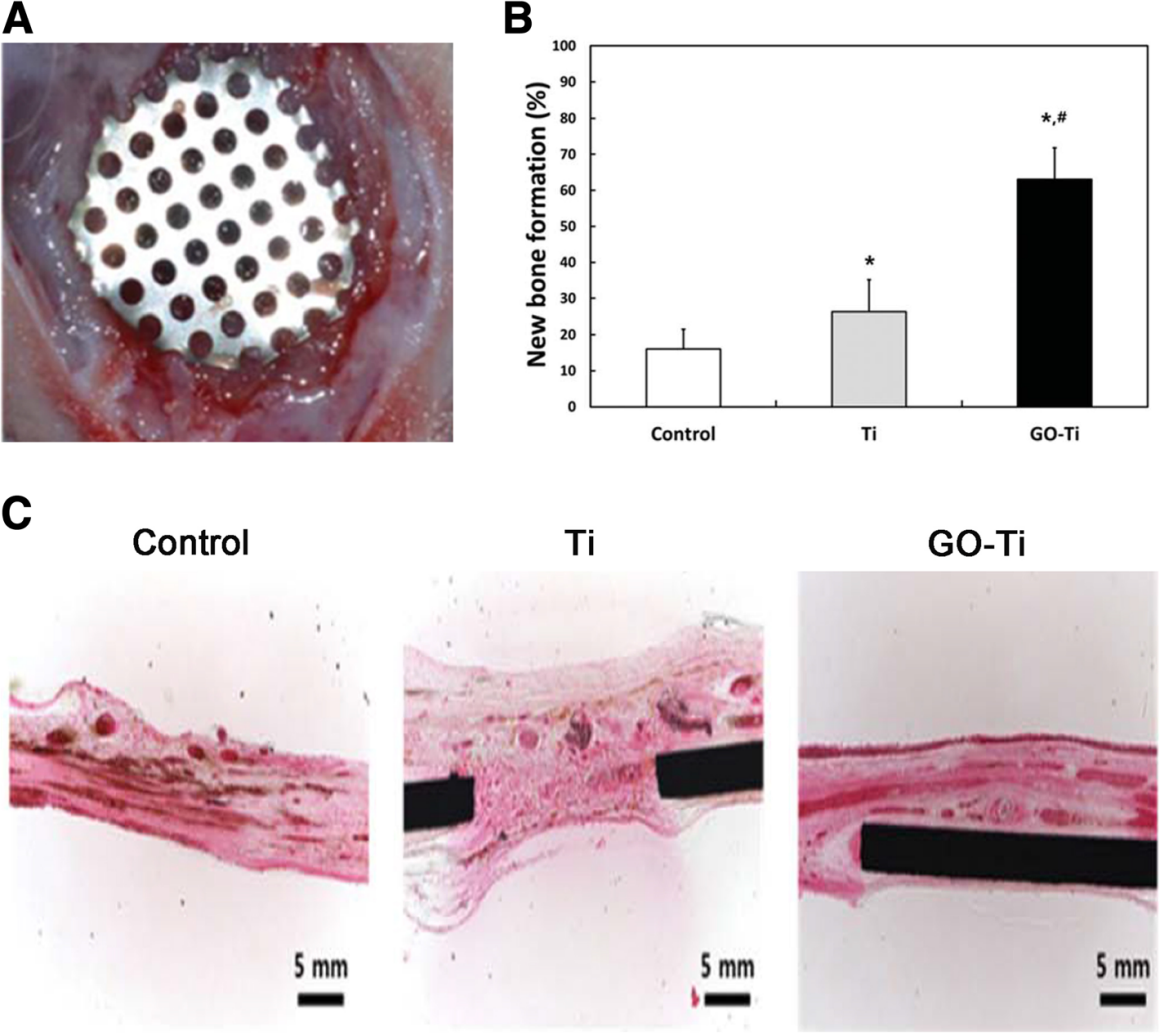
**a** The calvarial defects of rats were enclosed with a GO-Ti membrane. **b** New bone formation of the rat calvarial defects after the implantation of Ti or GO-Ti membrane at postoperative week 8. *p < 0.05 vs control; #*p* < 0.05 vs Ti. **c** Images of HE staining of the rat calvarial defects after the implantation of Ti or GO-Ti membrane at postoperative week 8. Reproduced from ref. [128] with permission from the Journal of Applied Spectroscopy Reviews

Osseointegration is a complicated biological process determined by the surface properties of implants [114]. The graphene-based coatings above all lack 3D morphology. The 3D porous surface structure of coating can mimic the special macrostructures of the nature bone tissues [115]. Qiu et al. first synthesized 3D porous graphene-based coating on the pure titanium plates (GO@Ti and rGO@Ti). Water contact angles showed super hydrophilic surfaces of GO@Ti and rGO@Ti. Surface wettability exerts great effect on the biocompatibility of materials, which is strongly related to biomolecules adsorption [121]. GO@Ti and rGO@Ti both showed the excellent cytocompability and the optimal capability of osteoinduction [39]. Morin and his co-workers even transferred single or double chemical vapor deposition (CVD) grown graphene coatings onto 3D objects with differences in 3D geometries and surface roughness, such us dental implant, locking compression plate and mandible plate (Fig. 6) [64]. CVD is a very stable coating fabrication method, with substrate-independent properties and versatile surface functionalization. Besides, surface active CVD coatings are good platforms for immobilizing biomolecules, which is very important to bone regeneration [122].

Overall, the strategy of applying graphene family materials as coating onto a surface is charming. Through currently available techniques or methods, such as CVD [123], electrochemical deposition [119], with diverse substrates (e.g. polymers, metals), graphene, and its derivatives can be obtained efficiently, with dimensions ranging from nanometer to macroscopic scales [120]. Then, graphene family nanomaterials can be transferred onto the substrate, either as 2D coatings/films/sheets or 3D porous structures of coating, to enable the binding of biomolecules, absorb the serum protein, and facilitate osteogenic differentiation of stem cells. But the different physical and chemical properties of the substrates and the type or frequent use of chemical inducers for osteogenic differentiation (e.g., dexamethasone, bone morphogenetic protein-2) that may cover up the effects exerted by graphene family materials alone [65]. Therefore, these methods still require to be well-directly improved and further studied.

### Graphene Family as an Additive in Guided Bone Membrane

Barrier membranes are standardly used in oral surgical procedures, applying in guided tissue regeneration (GTR) and guided bone regeneration (GBR), for the treatment of periodontal bone defects and peri-implant defects, as well as for bone augmentation [124, 125]. GBR is considered to be one of the most promising methods for bone tissue regeneration. The concept of GBR is using a non-resorbable or absorbable membrane serving as a barrier to prevent the ingrowth of soft connective tissue into the bone defect and offer a space to “guide” the bone reconstruction [126, 127]. An ideal GBR membrane should have excellent biocompatibility and mechanical property to promote the regeneration of bone tissues and prevent soft-tissue ingrowth. Ti membrane is a non-resorbable membrane with excellent mechanical properties for the stabilization of bone grafts. Park et al. fabricated GO-coated Ti (GO-Ti) membranes, with increased roughness and higher hydrophilicity. GO endowed the pure Ti membranes better biocompatibility and enhanced the attachment, proliferation, and osteogenesis of MC3T3-E1 in vitro. Moreover, GO-Ti membranes were implanted into rat calvarial defects (Fig. 6) and new bone formation significantly in full-thickness calvarial defects without inflammatory responses was observed [128].

However, non-resorbable membranes need to be removed by a second operation. Thus, a resorbable membrane is recommended owing to avoid a second intervention during operation, which can diminish the risk of infection and the loss of the regenerated bone. But the resorbable membranes made of collagen or chitosan usually has poor mechanical property. The addition of graphene family materials improves the weaknesses of resorbable membrane. For instance, De et al. attempted to prepare absorbable collagen membranes enriched with different concentrations of GO. The presence of GO on the membrane altered the mechanical features of the membrane, by conferring lower deformability, improving stiffness, and increasing roughness [129]. Tian et al. made 3D rGO (3D-rGO) porous films, which can accelerate cell viability and proliferation, as well as significantly enhanced ALP activity and osteogenic-related gene expressions [130].

Although pristine graphene is basically incompatible with organic polymer to form homogeneous composite, and even decrease the cell viability in some cases if the amount of graphene is excessive [131]. The incorporation of graphene family materials can enhance the bioactivity and mechanical properties of composite membranes. Because of the potent effects on altering mechanical drawbacks, stimulating osteogenic differentiation, and exhibiting superior bioactivity, graphene family material-modified membranes can be applied effectively to GBR.

### Graphene Family Materials as Drug Delivery System (DDS)

Due to their small size, intrinsic optical properties, large specific surface area, low cost, and useful noncovalent interactions with aromatic drug molecules, graphene family materials exhibit excellent efficacy as delivery vehicles of genes and biomolecules. Moreover, simple physisorption via π-π stacking, hydrogen bonding, and electrostatic interaction is able to assist in high drug loading of hydrophobic drugs without compromising potency or efficiency [38]. The therapeutic efficacy of drugs is always related to the drug delivery carrier, which should enable the loading of large doses, controlled release, and retention of the bioactivity of the therapeutic proteins [132]. At present, anticancer drugs, including doxorubicin [133–137], paclitaxel [138, 139], cisplatin [140], and methotrexate [141, 142] loaded by graphene family nanomaterials showed amazing cancerous effect for the selective killing of cancer cells.

For better bone regeneration, we sometimes need the help of osteogenic drug or macromolecular osteogenic protein. It was reported that the adsorbed drugs or loaded growth factors on graphene or its derivatives could enhance the osteogenic differentiation of cells due to the increased local concentration [143]. For example, simvastatin (SIM) chosen as a model drug was loaded on the 3D porous scaffolds, which were made of silk fibroin (SF) and GO. SIM is an inhibitor of the competitive 3-hydroxy-3-methyl coenzyme A (HMG-CoA) reductase [144]. The effects of SIM on bone formation are associated with an increase in the expression of bone morphogenetic protein-2 (BMP-2) mRNA and enhanced the vascular endothelial growth factor (VEGF) expression [145, 146]. SIM can release sustainedly (30 days), and the release rate was relevant to the GO content within the scaffolds. In vitro, compared with the blank scaffolds, the SF/GO/SIM showed better biocompatibility, and the cells cultured on them exhibited faster proliferation rate [147]. Dexamethasone (DEX) is an osteogenic drug for which can facilitate osseointegration. Jung et al. firstly loaded DEX on rGO-coated Ti by π-π stacking. The loading efficiency of DEX on rGO-Ti was 31% after drug loading for 24 h and only 10% of total loaded DEX was released for 7 days, indicating that the drug delivery system can induce a long-term stimulation of stem cells for osteogenic differentiation. The DEX/rGO-Ti significantly facilitated MC3T3-E1 cells growth and differentiation into osteoblasts [143]. Similarly, Ren et al. also employed the GO-Ti and rGO-Ti as drug vehicles to absorb DEX. The presence of DEX-GO and DEX-rGO helped to promote the cell proliferation and largely enhanced osteogenic differentiation [115]. The graphene family materials coating on Ti alloys with controlled drug delivery can stimulate and enhance cellular response around implant surface to reduce the osseointegration time, expected to be applied for various dental and biomedical applications [143].

**Fig. 7:**
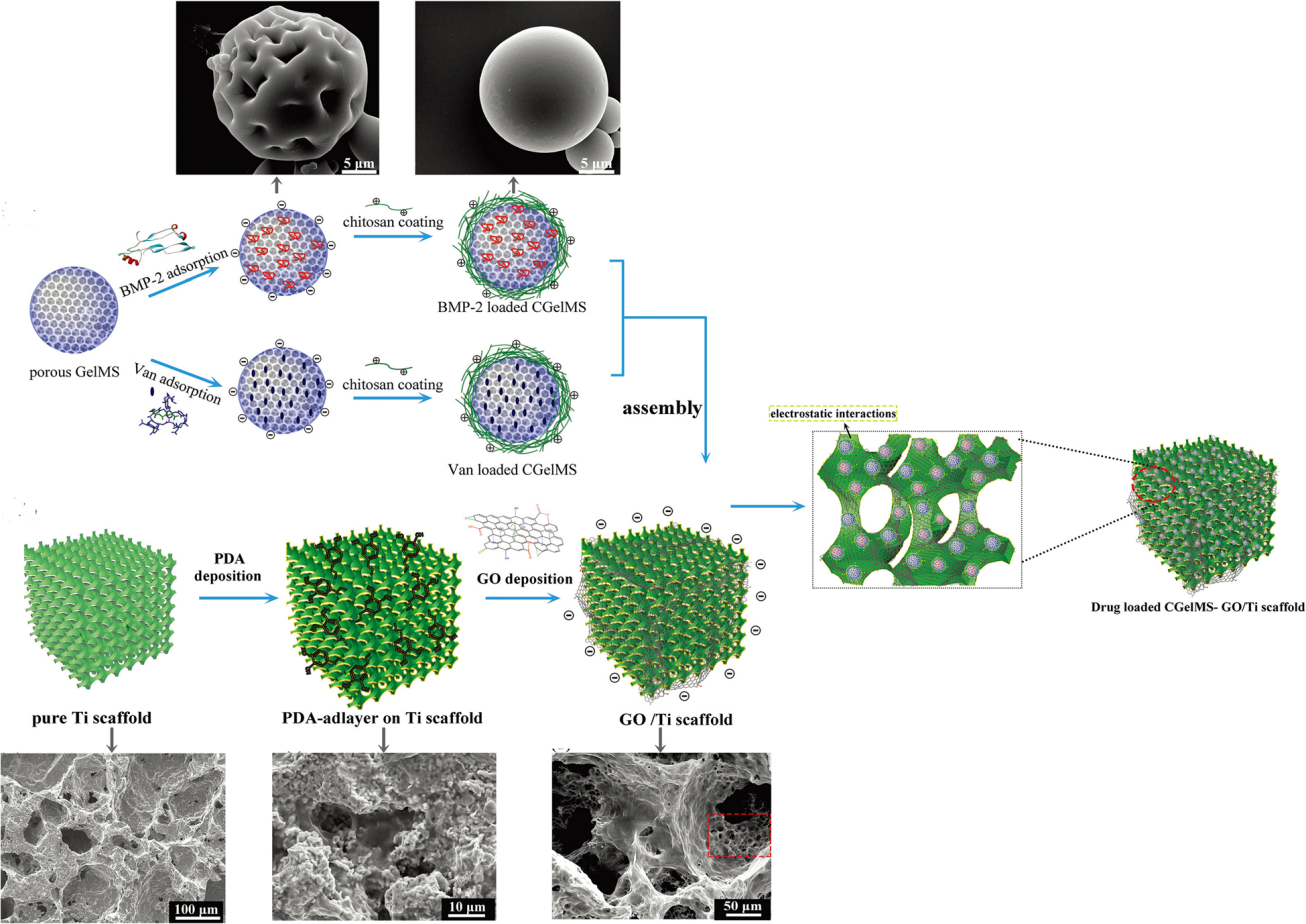
Schematics and scanning electron micrographs of the preparation the new GO/Ti scaffold: BMP2- and Van-loaded CGelMS were immobilized on the GO/Ti scaffold through electrostatic interactions between the functional groups of GO and CGelMS. Reproduced from ref. [149] with permission from the Journal of Biomaterials Science

Not only small molecular osteogenic drug, but also macromolecular proteins can be loaded by graphene family materials for bone regeneration. Bone morphogenetic proteins (BMPs) are the most potent osteoinductive protein for bone regeneration. Thus, BMP-2 was loaded on the surface of Ti/GO through π-π stacking and the interaction between negatively charged carboxylic groups at the edges of GO and positively charged amino acid residues of BMP-2 [132]. Ti/GO/BMP-2 exhibited the high loading and the sustained release of BMP-2 with preservation of its 3D conformational stability and bioactivity. In vitro, the capability of Ti/GO/BMP-2 is to enhance osteogenic differentiation of hBMSCs. In a mouse calvarial defect model, compared to Ti/BMP-2 implants, Ti/GO/BMP-2 implants around had much more extensive bone formation [132]. Xie et al. used GO-modified hydroxyapatite (HA) and GO-modified tricalcium phosphate (TCP) as an anchor for adsorbing BMP-encapsulated BSA- nanoparticles (NPs) respectively. The charge balance and BMP-2 sustained release capability of the new scaffolds synergistically improved BMSCs proliferation, differentiation, and bone regeneration in vivo [148]. Poor osteointegration and infection are the most serious complications leading to failures of Ti implantation [10]. Han et al. incorporated GO onto polydopamine (PDA)-modified Ti scaffolds. Then, BMP-2 and vancomycin (Van) were separately encapsulated into gelatin microspheres (GelMS). After that, drug-containing GelMS were loaded on GO/Ti scaffolds and anchored by the functional groups of GO (Fig. 7). The new scaffolds were endowed with dual functions of inducing bone regeneration and preventing bacterial infection [149]. Substance P (SP) is a highly conserved 11 amino acid neuropeptide [150], involved in many processes, such as the regulation of inflammation, wound healing, and angiogenesis, and it is expected to promote MSC recruitment to the implants [151]. Therefore, apart from BMP-2, La et al. added this peptide, SP, on the surface of GO-coated Ti. The dual delivery system via GO-coated Ti showed sustained release of BMP-2 and SP and the potential of SP for inducing migration of MSCs. In vivo, Ti/GO/SP/BMP-2 group showed the greater new bone formation in the mouse calvaria than Ti/GO/BMP-2 group may be due to the MSCs recruitment by SP to the implants [152].

Currently, more and more teams get down to designing new drug delivery system to improve the practical applications. The loading of large doses, controlled release, and retention of the bioactivity of the therapeutic proteins are still difficulty in research on drug delivery system.

## Conclusions

Studies on the graphene family materials on biological applications is emerging rapidly, especially their potential applications for bacteria inhibition and inducing stem cell osteogenic differentiation. Before their biological applications are considered for clinical trial, the biocompatibility of graphene family materials is of vital importance. However, the challenges exist and must be overcome. These challenges include a thorough understanding of the graphene-cell (or tissue, organ) interaction and cellular uptake mechanism as well as mechanism(s) of potential toxicity. We summarize and analyze several articles and conclude that the cytotoxicity and in vivo biocompatibility of graphene family materials are influenced by numerous factors, including surface functionalization, concentration, size, and shape. At low concentration, graphene family materials are cytocompatible, with little negative influence on cell morphology, viability, and proliferation. Furthermore, it was reported that graphene family materials with flat shapes having better biocompatibility, because the flat shape materials were expected to have minor interaction with the cellular membranes [47]. Although the different criteria were used to define the size scale and shape of graphene and its derivatives, it was true that nano-sized graphene family materials were much safer for biomedical applications [54]. Size-control synthesis of graphene family materials needs to be considered prudently in subsequent researches. Moreover, the major challenge for researchers lies in understanding how graphene family materials behave in complicated microenvironment and establishing the long-term biocompatibility of graphene and its derivatives. Thus, researchers should spare no efforts to keeping studying the bio-safety of graphene family materials in vivo, as well as in vitro, to further understand the intricate interaction between cells and the materials. Although some papers raise concerns about bio-safety, after better control of the modifying of graphene family materials during synthesis, the potential versatility that graphene family uniquely offers has made it a competitive candidate of option for biomedical applications.

On the one hand, a lot of researches have pointed out that graphene family materials possess the capability of bacteria inhibition, due to their functional chemical groups, sharp edges, and synergistic effect with other drugs. Besides, bone remolding and regenerating successfully in an infective bone defect area is challenging. Peri-implant infection and poor osseointegration are also major challenges we confront. The use of graphene family materials in the design and development of antimicrobial bone regeneration application will capture tremendous attention in the future.

On the other hand, lots of teams painstakingly did researches to design and fabricate the new strategies of applying graphene family materials in bone tissue engineering. 3D graphene-based scaffold is a promising biocompatible scaffold, which can enhance pre-osteoblasts or stem cells osteoblastic differentiation. Graphene family materials also can be added as a reinforced material aiming to strengthen the composite scaffold mechanical properties and improve physicochemical characterization. In addition, the strategy of applying graphene or its derivatives as coating onto a surface is charming, which is expected to possess the antibacterial activity and better osseointegration, especially the 3D coating. It has been generally hypothesized that the surface characteristics of graphene family materials including nanostructures, surface roughness, protein absorption ability, electrostatic interactions, and surface hydrophilicity, exert an enormous effect on the molecular pathways which control the fate of stem cells [39, 115]. The 3D structure of scaffold or coating allows nutrients to be freely delivered, which influences the biocompatibility of the graphene family. But the manufacturing method of 3D scaffold or coating is relatively difficult and complicated. However, with the rapidly development of the science and technology, the emerging of the 3D-printing method may overcome this problem and open an avenue for bone tissue regeneration.

Moreover, graphene family materials show great potential in GBR and DDS as well. Graphene family materials improve poor mechanical property of the resorbable membranes made of collagen or chitosan without compromising their intrinsic property. Osteogenic drug or macromolecular osteogenic protein can be adsorbed on graphene or its derivatives via π-π stacking, hydrogen bonding, and electrostatic interaction with high loading and good efficiency. Taking the varied merits into consideration, graphene family materials hold great potential to bone tissue regeneration.

Considering that many supreme properties graphene and its derivatives have, especially in vitro osteogenesis enhancing ability and excellent in vivo bone-forming ability, although they still have drawbacks, graphene family materials still are promising candidates used for bone regeneration applications.

## Abbreviations

ALP: Alkaline phosphatase
BMP-2: Bone morphogenetic protein-2
BMSC: Bone mesenchymal stem cells
CPC: Calcium phosphate cements
CVD: Chemical vapor deposition
CXYG: Carboxyl graphene
DEX: Dexamethasone
ECM: Extracellular matrix
GBR: Guided bone regeneration
GelMS: Gelatin microspheres
GNOs: Graphene nano-onions
GO: Graphene oxide
GONPs: Graphene oxide nanoplatelets
GONRs: Graphene oxide nanoribbons
GQDs: Graphene quantum dots
HA: Hydroxyapatite
HUVEC: Human umbilical vein endothelial cells
MC3T3-E1: A murine pre-osteoblastic cell line
MWNTs: Multi-walled carbon nanotubes
OPE: Oxygen plasma etching
PCL: Polycaprolactone
PDA: Polydopamine
PDLLA: Poly (d, l-lactic acid)
PDLSC: Periodontal ligament stem cells
PDMS: Polydimethylsiloxane
PEG: Polyethylene glycol
PET: Polyethylene terephthalate
PLA: Poly-lactic acid
PLGA: Poly-glycolic acid
PPY: Polypyrrole
rGO: Reduced graphene oxide
ROS: Reactive oxygen species
SIM: Simvastatin
SP: Substance P
SPS: Spark plasma sintering
Ti: Titanium
Van: Vancomycin
VEGF: Vascular endothelial growth factor
β-TCP: β-Tricalcium phosphate
